# Examination of Stigmatizing Language in the Electronic Health Record

**DOI:** 10.1001/jamanetworkopen.2021.44967

**Published:** 2022-01-27

**Authors:** Gracie Himmelstein, David Bates, Li Zhou

**Affiliations:** 1 Office of Population Research, Princeton University, Princeton, New Jersey; 2 Department of Medicine, University of California Los Angeles Health, Los Angeles; 3 Division of General Internal Medicine, Brigham and Women’s Hospital and Harvard Medical School, Boston, Massachusetts; 4 Department of Health Policy and Management, Harvard T. H. Chan School of Public Health, Boston, Massachusetts

## Abstract

**Importance:**

Stigmatizing language in the electronic health record (EHR) may alter treatment plans, transmit biases between clinicians, and alienate patients. However, neither the frequency of stigmatizing language in hospital notes, nor whether clinicians disproportionately use it in describing patients in particular demographic subgroups are known.

**Objective:**

To examine the prevalence of stigmatizing language in hospital admission notes and the patient and clinician characteristics associated with the use of such language.

**Design, Setting, and Participants:**

This cross-sectional study of admission notes used natural language processing on 48 651 admission notes written about 29 783 unique patients by 1932 clinicians at a large, urban academic medical center between January to December 2018. The admission notes included 8738 notes about 4309 patients with diabetes written by 1204 clinicians; 6197 notes about 3058 patients with substance use disorder by 1132 clinicians; and 5176 notes about 2331 patients with chronic pain by 1056 clinicians. Statistical analyses were performed between May and September 2021.

**Exposures:**

Patients’ demographic characteristics (age, race and ethnicity, gender, and preferred language); clinicians’ characteristics (gender, postgraduate year [PGY], and credential [physician vs advanced practice clinician]).

**Main Outcome and Measures:**

Binary indicator for any vs no stigmatizing language; frequencies of specific stigmatizing words. Linear probability models were the main measure, and logistic regression and odds ratios were used for sensitivity analyses and further exploration.

**Results:**

The sample included notes on 29 783 patients with a mean (SD) age of 46.9 (27.6) years. Of these patients, 1033 (3.5%) were non-Hispanic Asian, 2498 (8.4%) were non-Hispanic Black, 18 956 (63.6%) were non-Hispanic White, 17 334 (58.2%) were female, and 2939 (9.9%) preferred a language other than English. Of all admission notes, 1197 (2.5%) contained stigmatizing language. The diagnosis-specific stigmatizing language was present in 599 notes (6.9%) for patients with diabetes, 209 (3.4%) for patients with substance use disorders, and 37 (0.7%) for patients with chronic pain. In the whole sample, notes about non-Hispanic Black patients vs non-Hispanic White patients had a 0.67 (95% CI, 0.15 to 1.18) percentage points greater probability of containing stigmatizing language, with similar disparities in all 3 diagnosis-specific subgroups. Greater diabetes severity and the physician-author being less advanced in their training was associated with more stigmatizing language. A 1 point increase in the diabetes severity index was associated with a 1.23 (95% CI, .23 to 2.23) percentage point greater probability of a note containing stigmatizing language. In the sample restricted to physicians, a higher PGY was associated with less use of stigmatizing language overall (−0.05 percentage points/PGY [95% CI, −0.09 to −0.01]).

**Conclusions and Relevance:**

In this cross-sectional study, stigmatizing language in hospital notes varied by medical condition and was more often used to describe non-Hispanic Black patients. Training clinicians to minimize stigmatizing language in the EHR might improve patient-clinician relationships and reduce the transmission of bias between clinicians.

## Key Points

**Question:**

How frequently does stigmatizing language appear in the admission notes of patients who are hospitalized, and does the frequency vary by patients' medical conditions and race or ethnicity?

**Findings:**

In this cross-sectional study of 48 651 admission notes, 2.5% of all notes included stigmatizing language. Across all medical conditions studied, stigmatizing language appeared more frequently in notes written about non-Hispanic Black patients.

**Meaning:**

These findings suggest that improved conscientiousness and training around avoiding stigmatizing language in medical notes could improve health equity.

## Teaser

This cross-sectional study analyzes the prevalence of stigmatizing language in the electronic health records at a large academic medical center.

## Introduction

Health care clinicians spend many hours interacting with the electronic health record (EHR),^1,2^ which has become the primary means of communication between clinicians in the same practice, hospital, hospital network, and, increasingly, across systems via health information exchanges.^3^ With the 21st Century Cures Act’s implementation in April 2021, which mandates that clinicians offer patients access to EHR notes,^4^ the EHR has a new role as a mediator of relationships between clinicians and patients.

The EHR’s important role in clinician-clinician communications and clinician-patient relationships raises concerns about the use of stigmatizing language in medical records. Stigmas mark or signal that someone is less worthwhile and hence merits inferior treatment.^5^ Stigmas are not personal preferences but shared social constructions often communicated through language.^6^ Stigmatizing language generally takes 3 forms: (1) marking or labeling someone as other; (2) assigning responsibility (ie, blame); and (3) invoking danger or peril.^6^ All 3 forms of stigmatizing language may appear in the EHR. Some examples are familiar to clinicians: patients with substance use disorders labeled substance abusers; patients described as noncompliant or poorly controlled, emphasizing patient responsibility for their illness; and distressed patients being called belligerent or combative or implying purposeful efforts to endanger health care staff.

Stigmatizing language may compromise care by communicating discriminatory beliefs between clinicians. In a recent study,^7^ clinicians were more likely to use language indicating disbelief of patients in the medical records of Black patients. In vignette studies,^8,9^ clinicians were less likely to recommend treatment for patients labeled substance abusers than for those described as having substance use disorder. Clinicians reading vignettes about patients with sickle cell disease chose less aggressive pain management regimens and more often reported negative attitudes about patients when vignettes included stigmatizing language.^10^ Moreover, clinicians’ language use is important for building healthy clinician-patient relationships. Nationwide, approximately 60% of patients who are offered access to their EHRs viewed their records at least once.^11^ Stigmatizing language in records, when viewed by patients, may undermine trust,^12,13^ which may compromise health outcomes.^14^

Recently, some clinician and patient advocacy organizations and medical journals have published language guides to avoid and suggestions for preferred alternatives.^15,16^ However, much remains unknown about how frequently stigmatizing language appears in the EHR, which clinicians are most likely to use such language, and which patients’ notes are most likely to include it.

We used natural language processing to assess patterns of stigmatizing language use in the inpatient admission notes of all inpatients at an academic medical center and subgroups of patients with 3 conditions—diabetes, substance use disorder, and chronic pain. These conditions were selected because they are common among US inpatients (approximately 20% have a diagnosis of diabetes,^17^ 10% have a diagnosis of a substance use disorder,^18^ and 10% to 20% have a diagnosis of chronic pain^19,20^) and because they carry stigma.^21,22,23^ The conditions were also selected because literature exists on stigmatizing language in these conditions and because stigma’s adverse effects on care for these illnesses has been documented.^22,24,25^ We focused on admission notes because they are frequently read by other hospital staff and likely to influence how others view the patient. We assessed the prevalence of stigmatizing language and whether the use of such language was associated with patients or clinician demographic characteristics.

## Methods

The institutional review board (IRB) at Princeton University ceded review of this study to the IRB at Mass General Brigham, which approved it. Informed consent was waived because patient data were deidentified. This cross-sectional study follows the Strengthening the Reporting of Observational Studies in Epidemiology (STROBE) reporting guideline.

### Data and Processing

We analyzed free-text admission notes of all patients admitted to a large academic medical center in 2018. Each admission note was linked to *International Statistical Classification of Diseases and Related Health Problems, Tenth Revision *(*ICD-10*) codes enumerating the patient’s diagnoses and comorbidities and to their demographic characteristics, including race and ethnicity (based on designation in the HER, which is generally patient-reported, and included the choices Hispanic, non-Hispanic Asian, non-Hispanic Black, non-Hispanic White, or non-Hispanic other), age, gender, and preferred language. The text was also linked to the characteristics of the note’s author, including their credentials (dichotomized as physician vs advanced practice clinician [APC], a category that included physician assistants, nurse practitioners, nurse anesthetists, and nurse midwives); clinician post-graduate year (PGY), measured as years since receipt of a national provider identifier number; and clinician gender.

This study used race and ethnicity data as it was reported in the EHR, which may reflect self-report or may be determined by the administer who registered the patient. All patients who identified as Hispanic, regardless of race, were grouped into the Hispanic ethnicity category. Among the remaining patients, those identifying as Asian were grouped as non-Hispanic Asian, Black as non-Hispanic Black, White as non-Hispanic White, and those identifying as American Indian or Alaskan Native, Hawaiian, or Pacific Islander were grouped together in the category non-Hispanic other. Race and ethnicity were considered in this study because these social categories may make a patient vulnerable to being stigmatized.

We cleaned and parsed the free text of each note and tokenized the text into unigrams and bigrams (1- and 2-word units) for analysis. We assembled lists of stigmatizing language from published sources. For diabetes, we drew on guidelines from a task force convened by the Association of Diabetes Care and Education Specialists and the American Diabetes Association.^26^ For substance use, we drew on language guidelines established by the National Institute on Drug Abuse (NIDA).^27^ Stigmatizing language in chronic pain has significant overlap with stigmatizing language in substance use disorders, particularly language regarding opioid use.^24^ We defined stigmatizing language in chronic pain using the NIDA language guidelines for opioid use, supplemented by studies of stigmatizing language in pain.^10,28,29^ Using these same sources, we also assembled lists of nonstigmatizing language proposed as alternatives (eTable 1 in the Supplement). Table 1 displays the lists of stigmatizing terms; Table 2 presents illustrative examples of the context in which commonly used stigmatizing words appeared in the notes.

**Table 1.** Number of Uses of Stigmatizing Words and Phrases in the Hospital Admission Note

Stigmatizing language	Times each word or phrase appeared in admission notes, No.			
	Full sample (N = 48 651)	Diabetes (N = 8738)	Substance use disorder (N = 6197)	Chronic pain (N = 5176)
Note with any stigmatizing language, No. (%)	1197 (2.5)	599 (6.9)	209 (3.4)	37 (0.7)
Abuse^a^	5768	NA	3478	NA
Abuser	22	NA	11	NA
Abuses	3	NA	2	NA
Abusing	22	NA	12	NA
Addict	13	NA	10	NA
Addicted	18	NA	11	NA
Adherence	939	436	NA	NA
Adherent	707	183	NA	NA
Alcohol abuse	1963	NA	1112	NA
Argumentative	6	NA	1	NA
Been clean	27	NA	18	NA
Belligerent	8	NA	6	NA
Cheat	5	2	NA	NA
Cheating	7	1	NA	NA
Cheats	4	3	NA	NA
Combative	145	NA	NA	NA
Compliance	1460	608	NA	NA
Compliant	966	354	NA	NA
Control	14 634	3946	NA	NA
Controlled	16 153	5257	NA	NA
Controls	737	203	NA	NA
Degenerate	2	NA	0	NA
Depraved	0	NA	0	NA
Difficult patient	16	1	NA	NA
Drug problem	2	NA	2	1
Drug seeking	26	NA	NA	24
Fail	91	28	NA	NA
Failed	2847	600	NA	NA
Fails	263	74	NA	NA
Failure	25 899	8739	NA	NA
Fake	4	NA	NA	0
Faking	0	NA	NA	0
Habit	257	NA	77	NA
In denial	7	3	NA	NA
Junkie	0	NA	0	NA
Lifestyle disease	0	0	NA	NA
Malinger	0	NA	NA	0
Malingerer	1	NA	NA	0
Malingering	8	NA	NA	7
Malingers	1	NA	NA	0
Narcotic	660	NA	205	221
Narcotics	933	NA	290	295
Nonadherence	562	299	NA	NA
Nonadherent	98	27	NA	NA
Noncompliance	488	144	NA	NA
Noncompliant	147	104	NA	NA
Pill problem	0	NA	NA	NA
Pill seeking	0	NA	NA	NA
Pot head	0	NA	0	NA
Refuse	68	15	NA	NA
Refused	1293	389	NA	NA
Refuses	290	91	NA	NA
Secondary gain	15	NA	NA	11
Speedball	0	NA	0	NA
Strung out	0	NA	0	NA
Substance abuse	1080	NA	787	NA
Uncontrolled	890	416	NA	NA
Unmotivated	2	NA	NA	NA
Unwilling	78	21	1	NA
User	1678	NA	531	NA

Abbreviation: NA, not applicable.

^a^ Excluding substance abuse (which is tabulated separately).

**Table 2.** Examples of Stigmatizing Language in Context, by Condition

Condition	Examples
Diabetes	Patient failed to show up to endocrine follow up
	Noncompliant with insulin regimen
	Patient refused diabetic diet
Substance use disorder	Started on opioids for pain control and admits to becoming addicted to them
	Avoid narcotics given history of abuse
	He is a habitual cocaine user
Chronic pain	Questionable if hyperalgesia or drug seeking behavior
	Patient has numerous psychiatric diagnoses including malingering
	Concern for secondary gain given narcotic seeking behavior

Diagnoses of patients with diabetes, substance use disorder, and chronic pain were based on *ICD-10* codes. Because illness severity might influence stigmatizing language use, we also used *ICD-10* codes to assess the severity of diabetes and substance use disorder. For patients with diabetes, we calculated an adapted Diabetes Complications Severity Index (aDCSI), a validated tool for quantifying severity (range, 1-13) (eTable 2 in the Supplement).^30^ For patients with substance use disorder, we classified patients as: intoxicated without comorbid substance use disorder (score = 1); mild (score = 2); or moderate or severe (score = 3). We based these classifications on the crosswalk between *Diagnostic and Statistical Manual of Mental Disorders* (Fifth Edition) diagnoses and *ICD-10* codes available from the American Psychiatric Association.^31^ Additionally, we determined whether a substance use disorder of any severity was in remission using *ICD-10* codes.

### Statistical Analyses

We assigned each admission note a binary indicator of whether it included any stigmatizing terminology from the diagnosis-specific lists (ie, diabetes, substance use disorder, and chronic pain) for the full sample. For each of the 3 diagnosis-specific subsamples, we assigned binary indicators for the presence of any stigmatizing language related to that specific condition. We used regression models to assess the association between patient and clinician characteristics and any stigmatizing language in the whole sample or diagnosis-specific stigmatizing language in the subsamples. Our main models included a binary indicator for whether a clinician was a physician vs APC. All APCs in our sample were fully credentialed, but many physicians were trainees. Hence, to assess whether the use of stigmatizing language changed with additional training, we constructed separate models limited to physicians and medical students, which included years since medical school graduation (PGY) as a covariate, with negative values denoting pregraduation status (eg, −2 for third-year students). Additional models included an interaction term between race or ethnicity and preferred language to explore whether the relationship between patient race or ethnicity and use of stigmatizing language differed by patients’ preferred language. Models for diabetes controlled for severity using the aDCSI and diabetes type (1 vs 2). Models for substance use disorder included the severity score and an indicator of whether the substance use disorder was in remission.

We used multilevel models with random effects to account for the clustering of notes by clinician. In further analyses, we assessed clustering by patient, which was expected because of the low number of admission notes per patient; results were virtually identical to our main models’ and are not reported further. We report linear probability models for ease of interpretation.^32^ Logistic models yielded similar results, although the chronic pain model failed to converge (eTable 3 in the Supplement). We excluded pediatric patients and reran our models as a sensitivity analysis, which yielded nearly identical results (eTable 4 in the Supplement). We repeated our main models as a falsification test, substituting a binary indicator for the presence of any nonstigmatizing alternative language for the indicator of any stigmatizing terms (eTable 1 in the Supplement).

To illustrate differences in the use of specific stigmatizing words or phrases for each word or phrase, we (1) counted how many times it appeared in notes about non-Hispanic Black patients vs non-Hispanic White patients and divided those counts by the total count of other words in the notes for each group, generating the odds of each word appearing in notes about each group; and (2) calculated the ratio of these odds for non-Hispanic Black patients vs non-Hispanic White patients. These odds ratios have a similar interpretation as odds ratios produced from the more familiar logistic regression analyses. However, unlike the binary outcomes in logistic regression, our odds ratios are calculated using count data. In the Figure, we display these as logarithmic odds ratios (LORs), which have the advantage of visual symmetry. LORs may reflect random variation in word usage, particularly for infrequently used words when used in this context. Thus, we assess the statistical significance of these differences using the methods suggested by Monroe et al.^33^ In brief, these methods use a model-based approach with an informative Dirichlet prior probability distribution to generate a test statistic for determining the statistical significance of each odds ratio (eTable 5 in the Supplement). We repeated the analysis using word stems (eg, “abus” for “abusing,” “abuses,” and “abuser”) derived using the Porter2 stemming algorithm to examine whether differences were due to different forms of the same word stem.

Analyses used Python version 3.9 (Python) and R version 4.1 (R Project for Statistical Computing). A 2-sided *Z* test was used to determine LOR with significance set at *P* < .01. Statistical analyses were performed between May and September 2021.

## Results

In this study, the 29 783 patients had a mean (SD) of 46.9 (27.7) years and 17 334 (58.2) were female, 840 (2.8%) were Hispanic patients, 1033 (3.5%) non-Hispanic Asian patients, 2498 (8.4%) were non-Hispanic Black patients, 18 956 (63.6%) were non-Hispanic White patients, and 1394 (4.7%) were another race (including American Indian or Alaskan Native and Hawaiian or Pacific Islander), and 2939 (9.9%) preferred a language other than English (Table 3).

**Table 3.** Demographic Characteristics of Patients and Clinicians in the Sample

Characteristics	Participant, No. (%)			
	Whole sample	Diabetes	Substance use disorder	Chronic pain
Patient characteristics				
Patients, No.	29 783	4309	3058	2331
Age, mean (SD), y	46.9 (27.6)	66.8 (14.0)	55.4 (15.9)	61.3 (15.6)
Female patients	17 334 (58.2)	1950 (45.3)	1364 (44.6)	1323 (56.8)
Male patients	12 449 (41.8)	2359 (54.7)	1694 (55.4)	1008 (43.2)
Race and ethnicity				
Non-Hispanic Asian	1033 (3.5)	131 (3.0)	34 (1.0)	29 (1.2)
Non-Hispanic Black	2498 (8.4)	605 (14.0)	411 (13.4)	252 (10.8)
Hispanic	840 (2.8)	189 (4.4)	97 (3.2)	87 (3.7)
Non-Hispanic White	18 956 (63.6)	3012 (69.9)	2243 (73.3)	1806 (77.5)
Non-Hispanic other race^a^	1394 (4.7)	249 (5.8)	165(5.4)	115 (4.9)
Missing	5062 (17.0)	123 (2.9)	108 (3.5)	42(1.8)
Patient primary language other than English	2939 (9.9)	566 (13.1)	192 (6.3)	199 (8.5)
Spanish	1400 (4.7)	317 (7.4)	108 (3.5)	126 (5.4)
Arabic	260 (0.9)	58 (1.3)	11 (0.4)	21 (0.9)
Missing	383 (1.3)	51 (1.2)	34 (1.1)	22 (0.9)
Clinician characteristics				
Clinicians, No.	1932	1204	1132	1056
Advanced practice clinicians^b^	243 (12.6)	166 (13.8)	178 (15.7)	155 (14.7)
Female clinicians	1002 (51.9)	596 (49.5)	583 (51.5)	531 (50.3)
Male clinicians	930 (48.1)	608 (50.5)	549 (48.5)	525 (49.7)
Physician years since credentialed, mean (SD)	5.3 (4.7)	3.2 (3.7)	4.0 (4.0)	3.2 (3.7)
APC years since credentialed, mean (SD)	8.0 (3.9)	7.8 (4.0)	7.7 (4.6)	7.9 (3.8)

Abbreviation: APC, advanced practice clinician.

^a^ Non-Hispanic other race category includes American Indian or Alaskan Native, Hawaiian, and Pacific Islander patients.

^b^ Includes physician assistants, nurse practitioners, nurse anesthetists, and nurse midwives.

The sample consisted of 48 651 admission notes for 29 783 unique patients (mean [SD], 1.6 [1.21]; median [IQR], 1.0 [1] notes per patient) written by 1932 clinicians (mean [SD], 25.2 [71.1]; median [IQR], 9 [26] notes per clinician), including: 8738 notes about 4309 patients with diabetes written by 1204 clinicians; 6197 notes about 3058 patients with substance use disorder written by 1132 clinicians; and 5176 notes about 2331 patients with chronic pain written by 1056 clinicians. Race and ethnicity data were missing for 5062 admission notes in the overall sample, 4414 of the notes with this missing data were for newborns. Among notes regarding patients in the 3 diagnostic subgroups, race and ethnicity data were missing in less than 4% of records. Of authors of admission notes, 1689 (87.4%) were physicians, whose PGY ranged from −2 to 13 years; their mean (SD) PGY was 5.3 (4.7); APCs had been credentialed longer with a mean (SD) of 8.0 (3.9) years. Among authors, 1002 (51.9%) were female.

Stigmatizing language appeared in 1197 of all 48 651 notes (2.5%); diabetes-specific stigmatizing language appeared in 599 notes for patients with diabetes (6.9%); language stigmatizing substance use appeared in 209 notes for patients with substance use disorder (3.4%); 37 notes for patients with chronic pain included stigmatizing language regarding pain (0.7%) (Table 1).

Table 4 shows the multivariate associations between patient and clinician characteristics and stigmatizing language, accounting for clustering of notes by author. In the full sample, notes about non-Hispanic Black patients had a greater probability than those about non-Hispanic White patients of including stigmatizing language, a difference of 0.67 (95% CI, 0.15-1.18) percentage points, a 26.8% relative increase. Clustering because of a single clinician did not explain the variation in stigmatizing language use (intraclass correlation coefficient [ICC] = 0.00). Models limited to physician-authored notes yielded similar results and suggested that higher PGY was associated with less use of stigmatizing language overall (eTable 6 in the Supplement). In the sample restricted to physicians, higher PGY was associated with less use of stigmatizing language overall (−0.05 percentage points/PGY [95% CI, −0.09 to −0.01]). Including an interaction term between race or ethnicity and preferred language did not improve model fit (χ^2^ = 1.86, *P* = .76) (eTable 7 in the Supplement).

**Table 4.** Multilevel Linear Probability Models of the Presence of Any Stigmatizing Language in Admission Notes in the Full Sample and in Each of 3 Conditions

Factors^a^	Estimates (95% CI)			
	Full sample	Diabetes	Substance use disorder	Chronic pain
Intercept	0.0117 (0.0052 to 0.0181)	0.0718 (0.0408 to 0.1027)	0.0298 (−0.0006 to 0.0603)	0.0201 (0.0091 to 0.0311)
Patient age	0.0003 (0.0002 to 0.0004)	−0.0001 (−0.0005 to 0.0003)	−0.0004 (−0.0007 to −0.0001)	−0.0002 (−0.0004 to −0.0001)
Patient female	−0.0020 (−0.0054 to 0.0013)	−0.0114 (−0.0229 to 0.0001)	−0.0111 (−0.0210 to −0.0012)	0.0012 (−0.0039 to 0.0063)
Patient non-Hispanic Asian	−0.0116 (−0.0200 to −0.0032)	−0.0080 (−0.0413 to 0.0252)	−0.0228 (−0.0700 to 0.0243)	−0.0040 (−0.0247 to 0.0166)
Patient non-Hispanic Black	0.0067 (0.0015 to 0.0118**)**	0.0211 (0.0047 to 0.0374)	0.0216 (0.0077 to 0.0355**)**	0.0100 (0.0024 to 0.0177**)**
Patient Hispanic	0.0021 (−0.0059 to 0.0100**)**	0.0149 (−0.0119 to 0.0417)	0.0062 (−0.0192 to 0.0317**)**	0.0088 (−0.0048 to 0.0224
Patient non-Hispanic other race^b^	−0.0040 (−0.0125 to 0.0046**)**	−0.0012 (−0.0340 to 0.0315)	0.0158 (−0.0096 to 0.0412	−0.0083 (−0.0230 to 0.0063**)**
Preferred language other than English	0.0049 (−0.0017 to 0.0116**)**	−0.0104 (−0.0319 to 0.0111)	0.0159 (−0.0086 to 0.0403**)**	−0.0122 (−0.0241 to −0.0003**)**
Advanced practice clinician^c^	0.0018 (−0.0030 to 0.0066**)**	−0.0097 (−0.0266 to 0.0072)	0.0017 (−0.0122 to 0.0157**)**	−0.0013 (−0.0083 to 0.0057**)**
Female clinician	0.0002(−0.0037 to 0.0041**)**	−0.0002 (−0.0131 to 0.0127)	0.0074 (−0.0033 to 0.0182**)**	0.0024 (−0.0033 to 0.0081**)**
Diabetes Severity Index	NA	0.0123 (0.0023 to 0.0223)	NA	NA
Type 1 diabetes	NA	0.0056 (−0.0169 to 0.0282)	NA	NA
Substance use disorder severity	NA	NA	0.0112 (0.0016 to 0.0207**)**	NA
Substance use disorder in remission	NA	NA	−0.0103 (−0.0222 to 0.0016**)**	NA
Random effects	NA	NA	NA	NA
Within-author variance	0.03	0.07	0.03	0.01
Between-author variance	0.00	0.00	0.00	0.00
ICC	0.00	0.01	0.01	0.01
Authors, No.	1835	1191	1113	1043
Observations	40 098	8032	5627	4716

Abbreviations: ICC, interclass correlation coefficient; NA, not applicable.

^a^ Reference categories: patient male, patient non-Hispanic White, physician clinician, type 2 diabetes.

^b^ Non-Hispanic other race category includes American Indian or Alaskan Native and Hawaiian/Pacific Islander patients.

^c^ Advanced practice clinicians include nurse practitioners, physician assistants, nurse midwives and nurse anesthetists.

The LORs compare the frequency of the use of each stigmatizing word or phrase to describe non-Hispanic Black patients vs non-Hispanic White patients in the Figure. In the full sample, notes written about non-Hispanic Black patients had significantly greater odds than those about non-Hispanic White patients of including the words/phrases “nonadherence,” “belligerent,” “adherence,” “unwilling,” “compliance,” “abuser,” “uncontrolled,” “refused,” “drug seeking,” “abuse,” “refuses,” and “difficult patient.” LORs of word stems appear in the eFigure in the Supplement. The falsification test was not associated with racial patterns in use of nonstigmatizing, alternative language (eTable 8 in the Supplement).

**Figure.**
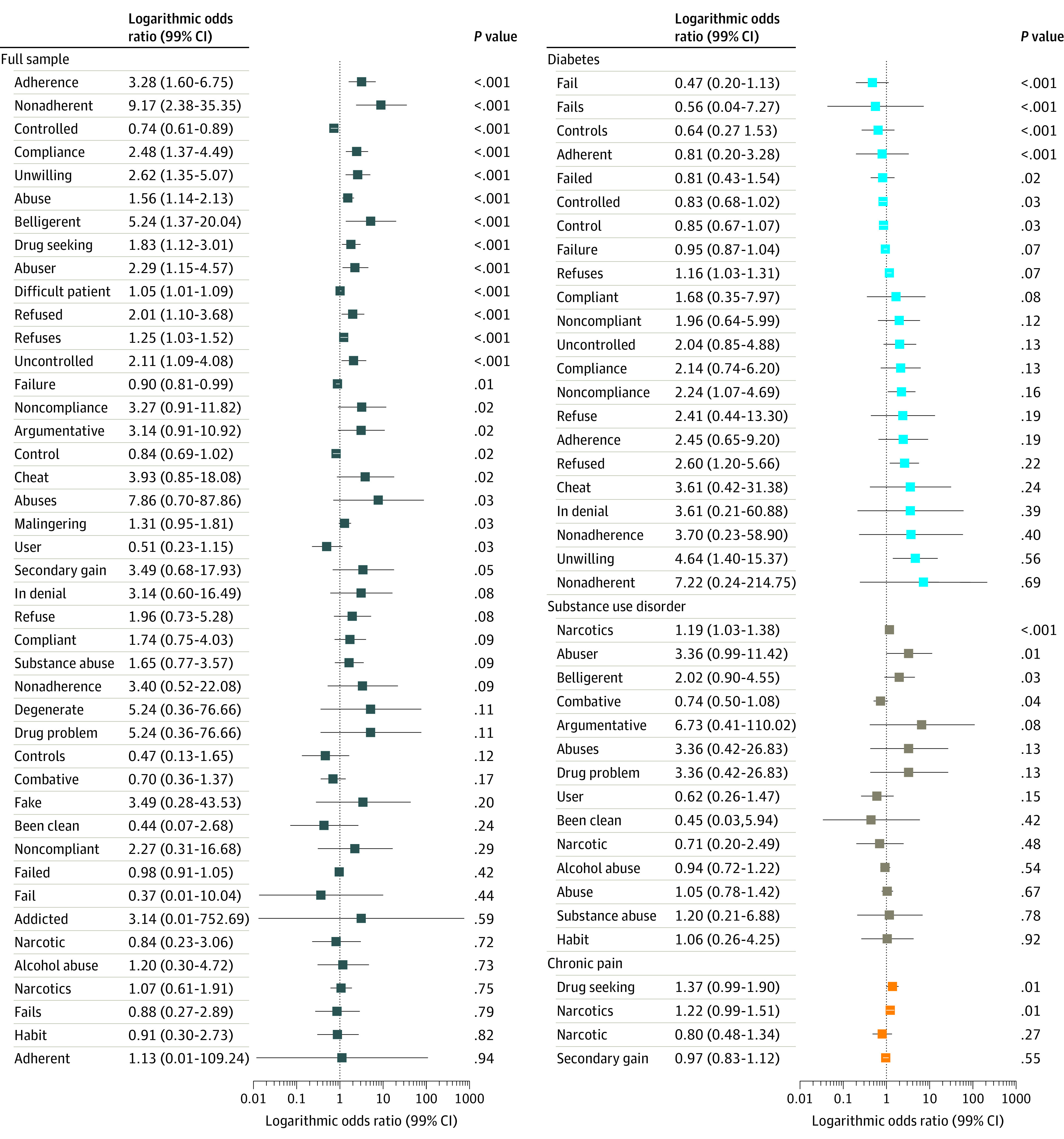
Logarithmic Odds Ratio for Stigmatizing Language in Whole Sample and by Condition Among Non-Hispanic Black Patients and Non-Hispanic White Patients Logarithmic odds ratios greater than 0 indicate language more commonly found in notes about non-Hispanic Black patients; those less than 0 indicate language more commonly found in notes about non-Hispanic White patients.

### Diabetes

Greater diabetes severity was associated with a higher probability of a note containing stigmatizing language (Table 4 and eTable 3 and eTable 4 in the Supplement). A 1 point increase in the diabetes severity index was associated with a 1.23 (95% CI, .23 to 2.23) percentage point greater probability of a note containing stigmatizing language. Notes written about non-Hispanic Black patients with diabetes were 2.11 percentage points (95% CI, 0.47-3.74) more likely to include stigmatizing language than notes written about non-Hispanic White patients (Table 4). Patient age, gender, preferred language, and other racial or ethnic categories were not associated with the probability of stigmatizing language, nor was any clinician characteristic. Notes for non-Hispanic Black patients had significantly greater odds of including the words “unwilling,” “refused,” “noncompliance,” and “refuses” (Figure).

### Substance Use and Chronic Pain

Relative to notes about non-Hispanic White patients, those about non-Hispanic Black patients had a 2.16 percentage point (95% CI 0.77, 3.55) greater probability of containing stigmatizing language (Table 4). As shown in Figure, the word “narcotics” had significantly greater odds of appearing in notes about non-Hispanic Black patients. Relative to notes written about non-Hispanic White patients with chronic pain, those about non-Hispanic Black patients had a 1.00 percentage point (95% CI, 0.24-1.77) greater probability of including stigmatizing language.

## Discussion

Stigmatizing language about diabetes, substance use disorder, or chronic pain appeared in 1 of 40 hospital admission notes and particularly frequently in the notes of patients with diabetes (1 in 15 notes). Across all conditions studied, notes about non-Hispanic Black patients more often included stigmatizing language than notes about non-Hispanic White patients. However, notes written by more experienced physicians with a higher PGY included less stigmatizing language than those written by less experienced physicians.

Although the stigmatizing language we assessed appeared infrequently, it has the potential to unnecessarily alienate patients and influence subsequent clinicians. We limited our list of stigmatizing words and phrases to those that have been well-documented in the literature, likely underestimating the total amount of stigmatizing language in the medical record. On the other hand, stigmatizing language is probably less common in notes about patients with less stigmatized conditions.

Our results augment a growing literature on stigmatizing language in the medical record. Previous researchers have assembled lists of stigmatizing words and phrases,^15,16^ identified common themes such as discrediting and disapproval in the negative language appearing in EHRs,^34^ and used vignettes to explore potential effects on treatment decisions.^9,10^ One study found that approximately 10% of patients who read their EHR felt judged or offended by their physician’s language.^12^ A recent study^7^ of physician outpatient notes found that notes about Black patients more often included language indicating disbelief of the patient. However, to our knowledge, ours is the first large-scale analysis quantifying the prevalence of stigmatizing language in the EHR and examining patient and clinician characteristics associated with its use.

Medical sociologists have noted that medical records are not just objective recordings of patients’ care but a venue where “…cultural assumptions, beliefs, and values are most directly displayed.”^35^ We found stigmatizing language appeared more frequently in notes about non-Hispanic Black patients, a finding not isolated to a few physicians in our sample. This is unsurprising given evidence that physicians (like the general US population) display pro-White and anti-Black attitudes on tests of implicit bias,^36^ and that this racism adversely affects the care provided to patients of color.^37,38^

Beyond likely reflecting physicians’ racial biases, the codification of stigma regarding Black patients in the EHR raises 2 concerns. Because the medical record may transmit stigma, stigmatizing language in notes may magnify the adverse health consequences of stigma imposed by racism in other venues.^39^ Furthermore, the history of medical experimentation and physician mistreatment of Black patients has undermined the trust of many racial and ethnic minority individuals in the medical system,^40,41^ which may cause avoidance of vaccines and other care.^42,43^ As patients gain access to their records, the disproportionate use of stigmatizing language in notes for Black patients risks deepening patients’ distrust and undermining efforts to promote racial equity in care.

### Limitations

This study has limitations. While we compiled lists of stigmatizing language from existing literature, no consensus exists about what language is stigmatizing, and many stigmatizing terms have not been linked to substandard care. Our dictionary-based natural language processing approach allowed us to identify the frequencies and patterns of stigmatizing language use, but some instances of stigmatizing language we captured would not be viewed as stigmatizing in context. Moreover, it may be challenging for physicians to accurately document patients’ care without the use of stigmatizing words, such as nonadherence, and many things that should be documented in patients' records (eg, substance use disorders) might be somewhat stigmatizing even if written in the most respectful way possible. Conversely, we likely missed some instances of stigmatizing language.

We used racial categories and language preferences recorded in the EHR. While these may include inaccuracies, studies suggest they generally accord with patients’ self-reports.^44^ Because race and ethnicity data were missing in the records of many newborns our findings cannot be applied to them.

Our data did not include measures of socioeconomic status (SES), precluding analysis of whether differences in SES play a role in the race-based disparities we observed. Untangling the roles of race and SES is particularly complex because racism is associated with low SES. Exploration of the relationships between patient race and ethnicity, SES, and the use of stigmatizing language is an important area for future study.

We found evidence that stigmatizing language appeared more commonly in notes of patients with more severe illness, defined using *ICD-10* codes. However, these codes are assigned based on clinicians’ documentation, which might differ according to patients’ race, potentially biasing our analysis. However, we know of no evidence that *ICD-10* coding differs by patient race. While the notes in the sample were written by clinicians trained at diverse institutions, our study encompassed inpatient admission notes from a single institution, which might differ from the language used at other hospitals or in outpatient settings.

## Conclusions

Our findings suggest that stigmatizing language appears in patients’ EHR admission notes, varies by medical condition, and is more often used to describe non-Hispanic Black than non-Hispanic White patients. Therefore, efforts to understand and minimize the use of stigmatizing language might improve patients’ care and their trust in their clinicians.

## Notes

Supplement. **eTable 1. **Number of Uses of Alternatives to Stigmatizing Words and Phrases in the Hospital Admission Note **eTable 2. **Adapted Diabetes Complications Severity Index (aDCSI) Score Rubric **eTable 3. **Simplified Multilevel Logistic Models of Having Any Stigmatizing Language in Note, with Odds Ratios and 95% CIs by Condition **eTable 4.** Multilevel Linear Probability Models of the Presence of Any Stigmatizing Language in Admission Notes for Adult Patients Age 18 Years or Older **eTable 5.** Significant Log Odds Ratios, Odds Ratios, and Weighted Log Odds Ratios of Words in Notes About Non-Hispanic Black vs Non-Hispanic White Patients **eTable 6.** Multilevel Linear Probability Models of the Presence of Any Stigmatizing Language in Admission Notes Written by Physicians in the Full Sample and in Each of 3 Conditions **eTable 7. **Multilevel Linear Probability Models of the Presence of Any Stigmatizing Language in Admission Note in the Full Sample and in Each of 3 Conditions, with Interaction Terms **eTable 8. **Falsification Analysis—Multilevel Linear Probability Modelsof the Presence of Any Alternative to Stigmatizing Language in Admission Note in the Full Sample and in Each of 3 Conditions **eFigure.** Log Odds Ratios (non-Hispanic Black/non-Hispanic White) For WordStems of Stigmatizing Language, in Whole Sample and by Condition
