# Supplementary material for: Examination of Stigmatizing Language in the Electronic Health Record
Source: JAMA Netw Open. 2022 Jan 27;5(1):e2144967. doi: 10.1001/jamanetworkopen.2021.44967 (PMC8796019; doi:10.1001/jamanetworkopen.2021.44967)
Supplement: Supplement. — eTable 1. Number of Uses of Alternatives to Stigmatizing Words and Phrases in the Hospital Admission Note eTable 2. Adapted Diabetes Complications Severity Index (aDCSI) Score Rubric eTable 3. Simplified Multilevel Logistic Models of Having Any Stigmatizing Language in Note, with Odds Ratios and 95% CIs by Condition eTable 4. Multilevel Linear Probability Models of the Presence of Any Stigmatizing Language in Admission Notes for Adult Patients Age 18 Years or Older eTable 5. Significant Log Odds Ratios, Odds Ratios, and Weighted Log Odds Ratios of Words in Notes About Non-Hispanic Black vs Non-Hispanic White Patients eTable 6. Multilevel Linear Probability Models of the Presence of Any Stigmatizing Language in Admission Notes Written by Physicians in the Full Sample and in Each of 3 Conditions eTable 7. Multilevel Linear Probability Models of the Presence of Any Stigmatizing Language in Admission Note in the Full Sample and in Each of 3 Conditions, with Interaction Terms eTable 8. Falsification Analysis—Multilevel Linear Probability Modelsof the Presence of Any Alternative to Stigmatizing Language in Admission Note in the Full Sample and in Each of 3 Conditions eFigure. Log Odds Ratios (non-Hispanic Black/non-Hispanic White) For WordStems of Stigmatizing Language, in Whole Sample and by Condition [file jamanetwopen-e2144967-s001.pdf]

Supplemental Online Content

Himmelstein G, Bates D, Zhou L. Examination of stigmatizing language in the electronic health record. *JAMA Netw Open*. 2022;5(1):e2144967. doi:10.1001/jamanetworkopen.2021.44967

eTable 1. Number of Uses of Alternatives to Stigmatizing Words and Phrases in the Hospital Admission Note
eTable 2. Adapted Diabetes Complications Severity Index (aDCSI) Score Rubric
eTable 3. Simplified Multilevel Logistic Models of Having Any Stigmatizing Language in Note, with Odds Ratios and 95% CIs by Condition
eTable 4. Multilevel Linear Probability Models of the Presence of Any Stigmatizing Language in Admission Notes For Adult Patients Age 18 Years or Older
eTable 5. Significant Log Odds Ratios, Odds Ratios, and Weighted Log Odds Ratios of Words in Notes About Non-Hispanic Black vs Non-Hispanic White Patients
eTable 6. Multilevel Linear Probability Models of the Presence of Any Stigmatizing Language in Admission Notes Written by Physicians in the Full Sample and in Each of 3 Conditions
eTable 7. Multilevel Linear Probability Models of the Presence of Any Stigmatizing Language in Admission Note in the Full Sample and in Each of 3 Conditions, with Interaction Terms
eTable 8. Falsification Analysis—Multilevel Linear Probability Models of the Presence of Any Alternative to Stigmatizing Language in Admission Note in the Full Sample and in Each of 3 Conditions
eFigure. Log Odds Ratios (Non-Hispanic Black/Non-Hispanic White) For WordStems of Stigmatizing Language, in Whole Sample and by Condition

This supplemental material has been provided by the authors to give readers additional information about their work.

© 2022 Himmelstein G et al. *JAMA Network Open.*

eTable 1. No. of Uses of Alternatives to Stigmatizing Words and Phrases in the Hospital Admission Note

**Stigmatizing Language in Full Sample** N=48,651 Notes		**Stigmatizing Language in Diabetes** N=8,738 Notes		**Stigmatizing Language in Substance Use** N=6,197 Notes		**Stigmatizing Language in Chronic Pain** N=5,176 Notes	
**Percent of Notes with Any Stigmatizing Language**							
5.4%		12.4%		2.3%		6.1%	
**Total Number of Times Each Word or Phrase Appeared in Notes**							
	**#**		**#**		**#**		**#**
Active use	12	As prescribed	172	Active use	11	Opiate	361
Actively used	1	Checked	602	Actively used	1	Opioid	782
Actively uses	1	Checks	655	Actively uses	1	Somatic symptom	3
Actively using	15	Engaged	59	Actively using	13	Somatoform	4
As prescribed	497	Engagement	12	Drug addiction	8		
Checked	2673	Engages	38	Harmful use	0		
Checks	2007	Involved	1006	Hazardous use	0		
Drug addiction	14	Involvement	1382	Misuse	44		
Engaged	223	Managed	3054	Misuses	1		
Engagement	55	Management	6350	Urine toxicology	191		
Engages	151	Manages	109	Use disorder	1598		
Harmful use	0	Participate	327	Utox	791		
Hazardous use	0	Participated	61				
Involved	4686	Participates	7				
Involvement	7145	Participation	63				
Managed	9648	Take	64063				
Management	26430	Takes	2074				
Manages	337	Took	1093				
Misuse	337						
Misuses	89						
Opiate	1						
Opioid	928						
Participate	1223						
Participated	239						
Participates	34						
Participation	253						
Somatic symptom	3						
Somatoform	5						
Take	215022						
Takes	7565						
Took	4539						
Urine toxicology	584						
Use disorder	2008						
Utox	1453						

© 2022 Himmelstein G et al. *JAMA Network Open.*

eTable 2. Adapted Diabetes Complications Severity Index (aDCSI) Score Rubric

Diagnosis	ICD-10 Code	Points
**Retinopathy**		
Diabetes Mellitus due to underlying conditions with ophthalmic complications	E08.3x excluding E08.34x and E08.35x	1
Drug or chemical induced diabetes mellitus with ophthalmic complications	E09.3x excluding E09.34x and E09.35x	1
Type 1 diabetes mellitus with ophthalmic complications	E10.3x excluding E10.34x and E10.35x	1
Type 2 diabetes mellitus with ophthalmic complications	E11.3x excluding E11.34x and E11.35x	1
Other specified diabetes mellitus with ophthalmic complications	E13.3x excluding E13.34x and E13.35x	1
Background retinopathy and retinal vascular changes	H35.0x	1
Cystoid macular degeneration	H35.35x	1
Retinal hemorrhage	H35.6x	1
Other specified retinal disorders	H35.8x	1
Unspecified retinal disorder	H35.9	1
Retinal detachments and breaks	H33.x	2
Diabetes Mellitus due to underlying conditions with severe non-proliferative diabetic retinopathy	E08.34x	2
Diabetes Mellitus due to underlying conditions with proliferative diabetic retinopathy	E08.35x	2
Drug or chemical induced diabetes mellitus with severe non-proliferative diabetic retinopathy	E09.34x	2
Drug or chemical induced diabetes mellitus with proliferative diabetic retinopathy	E09.35x	2
Type 1 diabetes mellitus with severe non-proliferative diabetic retinopathy	E10.34x	2
Type 1 diabetes mellitus with proliferative diabetic retinopathy	E10.35x	2
Type 2 diabetes mellitus with severe non-proliferative diabetic retinopathy	E11.34x	2
Type 2 diabetes mellitus with proliferative diabetic retinopathy	E11.35x	2
Other specified diabetes mellitus with severe non-proliferative diabetic retinopathy	E13.34x	2
Other specified diabetes mellitus with proliferative diabetic retinopathy	E13.35x	2
Blindness and low vision	H54.x	2
Vitreous hemorrhage	H43.1x	2
**Retinopathy Score**		**/2**
**Nephropathy**		
Diabetes mellitus due to underlying condition with diabetic nephropathy	E08.21	1
Diabetes mellitus due to underlying condition with diabetic chronic kidney disease	E08.22	1
Diabetes mellitus due to underlying condition with other diabetic kidney complication	E08.29	1
Drug or chemical induced diabetes mellitus with diabetic nephropathy	E09.21	1
Drug or chemical induced diabetes mellitus with diabetic chronic kidney disease	E09.22	1
Drug or chemical induced diabetes mellitus with other diabetic kidney complication	E09.29	1
Type 1 diabetes mellitus with diabetic nephropathy	E10.21	1
Type 1 diabetes mellitus with diabetic chronic kidney disease	E10.22	1
Type 1 diabetes mellitus with other diabetic kidney complication	E10.29	1
Type 2 diabetes mellitus with diabetic nephropathy	E11.21	1
Type 2 diabetes mellitus with diabetic chronic kidney disease	E11.22	1
Type 2 diabetes mellitus with other diabetic kidney complication	E11.29	1

© 2022 Himmelstein G et al. *JAMA Network Open.*

Diagnosis	ICD-10 Code	Points
Other specified diabetes mellitus with diabetic nephropathy	E13.21	1
Other specified diabetes mellitus with diabetic chronic kidney disease	E13.22	1
Other specified diabetes mellitus with other diabetic kidney complication	E13.29	1
Acute nephritic syndrome	N00.x	1
Nephrotic syndrome	N04.x	1
Chronic nephritic syndrome	N03.x	1
Unspecified nephritic syndrome	N05.x	1
Chronic Kidney Disease, Stage 1	N18.1	1
Chronic Kidney Disease, Stage 2	N18.2	1
Chronic Kidney Disease, Stage 3	N18.3	1
Chronic Kidney Disease, unspecified	N18.9	1
Chronic Kidney Disease, Stage 4	N18.4	2
Chronic Kidney Disease, Stage 5	N18.5	2
End stage renal disease	N18.6	2
Unspecified kidney failure	N19	2
**Nephropathy Score**		**/2**
**Neuropathy**		
Diabetes mellitus due to underlying condition with neurological complications	E08.4x	1
Drug or chemical induced diabetes mellitus with neurological complications	E09.4x	1
Type 1 diabetes mellitus with neurological complications	E10.4x	1
Type 2 diabetes mellitus with neurological complications	E11.4x	1
Other specified diabetes mellitus with neurological complications	E13.4x	1
Other [than carotid sinus syncope] idiopathic peripheral autonomic neuropathy	G90.09	1
Other disorders of autonomic nervous system	G90.8	1
Disorder of the autonomic nervous system, unspecified	G90.9	1
Autonomic neuropathy in diseases classified elsewhere	G99.0	1
Mononeuropathies of upper limb	G56.x	1
Mononeuropathies of lower limb	G57.x	1
Hereditary and idiopathic neuropathy, unspecified	G60.9	1
Myasthenic syndromes in other diseases classified elsewhere	G73.3	1
Carotid sinus syncope	G90.01	1
Paralytic strabismus	H49.x	1
Orthostatic hypotension	I95.1	1
Gastroparesis	K31.84	1
Functional diarrhea	K59.1	1
Neuromuscular dysfunction of bladder, unspecified	N31.9	1
Charcôt's joint	M14.6x	1
Injury to cranial nerve	S04.x	1
**Neuropathy Score**		**/1**
**Cerebrovascular**		
Transient cerebral ischemic attacks and related syndromes	G45.x	1
Nontraumatic intracerebral hemorrhage	I61.x	2
Cerebral infarction	I63.x	2
Occlusion and stenosis of precerebral arteries, not resulting in cerebral infarction	I65.x	2
Occlusion and stenosis of cerebral arteries, not resulting in cerebral infarction	I66.x	2
Acute cerebrovascular insufficiency	I67.81	2
**Cerebrovascular Score**		**/2**
**Cardiovascular**		

© 2022 Himmelstein G et al. *JAMA Network Open.*

Diagnosis	ICD-10 Code	Points
Other acute ischemic heart disease	I24.x	1
Angina pectoris	I20.x	1
Chronic ischemic heart disease	I25.x,<br>excluding I25.2	1
Atherosclerosis	I70.x,<br>excluding I70.25 and I70.26x	1
STEMI and NSTEMI	I21.x	2
Subsequent STEMI and NSTEMI	I22.x	2
Complications following STEMI and NSTEMI	I23.x	2
Old myocardial infarction	I25.2	2
Atrial fibrillation and flutter	I48.x	2
Cardiac arrest	I46.x	2
Paroxysmal tachycardia	I47.x	2
Other cardiac arrhythmias	I49.x	2
Heart failure	I50.x	2
Atherosclerosis of native arteries of the extremities with ulceration	I70.25	2
Atherosclerosis of native arteries of the extremities with gangrene	170.26x	2
Aortic aneurysm/dissection	I71.x	2
**Cardiovascular Score**		**/2**
**Peripheral Vascular Disease**		
Diabetes mellitus due to underlying condition with diabetic peripheral angiopathy without gangrene	E08.51	1
Diabetes mellitus due to underlying condition with other circulatory complications	E08.59	1
Diabetes mellitus due to underlying condition with diabetic foot ulcer	E08.621	1
Drug or chemical induced diabetes mellitus with diabetic peripheral angiopathy without gangrene	E09.51	1
Drug or chemical induced diabetes mellitus with other circulatory complications	E09.59	1
Drug or chemical induced diabetes mellitus with diabetic foot ulcer	E09.621	1
Type 1 diabetes mellitus with diabetic peripheral angiopathy without gangrene	E10.51	1
Type 1 diabetes mellitus with other circulatory complications	E10.59	1
Type 1 diabetes mellitus with diabetic foot ulcer	E10.621	1
Type 2 diabetes mellitus with diabetic peripheral angiopathy without gangrene	E11.51	1
Type 2 diabetes mellitus with other circulatory complications	E11.59	1
Type 2 diabetes mellitus with diabetic foot ulcer	E11.621	1
Other specified diabetes mellitus with diabetic peripheral angiopathy without gangrene	E13.51	1
Other specified diabetes mellitus with other circulatory complications	E13.59	1
Other specified diabetes mellitus with diabetic foot ulcer	E13.621	1
Atherosclerosis of native arteries of extremities with intermittent claudication	I70.21x	1
Other specified peripheral vascular diseases	I73.89	1
Peripheral vascular disease, unspecified	I73.9	1
Open wound of foot	S91.3x	1
Gas gangrene	A48.0	2
Embolism and thrombosis of arteries of the lower extremities	I74.3	2
Non-pressure chronic ulcer of lower limb, not elsewhere classified	L97.x	2
Diabetes mellitus due to underlying condition with diabetic peripheral angiopathy, with gangrene	E08.52	2

© 2022 Himmelstein G et al. *JAMA Network Open.*

Diagnosis	ICD-10 Code	Points
Drug or chemical induced diabetes mellitus with diabetic peripheral angiopathy, with gangrene	E09.52	2
Type 1 diabetes mellitus with diabetic peripheral angiopathy, with gangrene	E10.52	2
Type 2 diabetes mellitus with diabetic peripheral angiopathy, with gangrene	E11.52	2
Other specified diabetes mellitus with diabetic peripheral angiopathy, with gangrene	E13.52	2
Gangrene, not elsewhere classified	I96	2
**Peripheral Vascular Disease Score**		**/2**
**Metabolic**		
Diabetes mellitus due to underlying condition with hyperosmolarity, with coma	E08.01	2
Drug or chemical induced diabetes mellitus with hyperosmolarity, with coma	E09.01	2
Type 1 diabetes mellitus with hyperosmolarity, with coma	E10.01	2
Type 2 diabetes mellitus with hyperosmolarity, with coma	E11.01	2
Other specified diabetes mellitus with hyperosmolarity, with coma	E13.01	2
Diabetes mellitus due to underlying condition with ketoacidosis	E08.1x	2
Drug or chemical induced diabetes mellitus with ketoacidosis	E09.1x	2
Type 1 diabetes mellitus with ketoacidosis	E10.1x	2
Type 2 diabetes mellitus with ketoacidosis	E11.1x	2
Other specified diabetes mellitus with ketoacidosis	E13.1x	2
Diabetes mellitus due to underlying condition with hypoglycemia with coma	E08.641	2
Drug or chemical induced diabetes mellitus with hypoglycemia with coma	E09.641	2
Type 1 diabetes mellitus with hypoglycemia with coma	E10.641	2
Type 2 diabetes mellitus with hypoglycemia with coma	E11.641	2
Other specified diabetes mellitus with hypoglycemia with coma	E13.641	2
**Metabolic Score**		**/2**
**Total aDCSI Score**		**/13**

© 2022 Himmelstein G et al. *JAMA Network Open.*

eTable 3. Simplified[a] Multilevel Logistic Models[b] of Having Any Stigmatizing Language in Note, with Odds Ratios and 95% CIs by Condition[c]

	Full Sample		Diabetes		Substance Use	
Predictors	*Odds Ratios*	*CI*	*Odds Ratios*	*CI*	*Odds Ratios*	*CI*
(Intercept)	0.01	0.01,0.02	0.07	0.04,0.11	0.02	0.01,0.06
Patient Age	1.01	1.01,1.01	1.00	0.99,1.01	0.99	0.98,1.00
Patient Female	0.92	0.81,1.05	0.84	0.70,1.00	0.72	0.53,0.99
Patient Non-Hispanic Black	1.28	1.05,1.55	1.35	1.07,1.71	1.73	1.18,2.51
Patient Hispanic	1.08	0.78,1.49	1.28	0.84,1.94	1.21	0.60,2.47
Patient Non-Hispanic Other Race[d]	0.64	0.46,0.88	0.92	0.62,1.37	1.25	0.65,2.37
Preferred Language Other than English	1.19	0.92,1.54	0.84	0.59,1.19	1.43	0.75,2.71
Advanced Practice Provider (APP)	1.10	0.91,1.33	0.85	0.65,1.13	1.07	0.69,1.64
Female Provider	1.00	0.86,1.17	0.99	0.81,1.22	1.24	0.88,1.75
Diabetes Severity Index (aDCSI)			1.20	1.03,1.41		
Type 1 Diabetes Mellitus			1.06	0.76,1.49		
Substance Use Severity					1.40	1.02,1.92
Substance Use Disorder in Remission					0.76	0.54,1.08
**Random Effects**						
σ^2	3.29		3.29		3.29	
τ_{00}	0.19 $_{author}$		0.19 $_{author}$		0.38 $_{author}$	
ICC	0.05		0.05		0.10	
N	1835 $_{author}$		1191 $_{author}$		1113 $_{author}$	
Observations	40098		8032		5627	

[a]Simplified by including Patient Non-Hispanic Asian in Patient Non-Hispanic Other Race. This was done to achieve model convergence.
[b] Reference Categories: patient male, patient Non-Hispanic White, physician provider, type II diabetes mellitus
[c]Chronic pain excluded as model over-specified and failed to converge, even after simplification.
[d]Non-Hispanic Other Race category includes Native American and Hawaiian/Pacific Islander patients
Advanced practice providers include nurse practitioners, physician assistants, nurse midwives and nurse anesthetists
aDCSI= adapted diabetes complication severity index
σ^2 = Within author variance, τ_{00} = Between author variance
ICC = interclass correlation coefficient, which indicates that share of the variance explained by single providers who authored multiple note

© 2022 Himmelstein G et al. *JAMA Network Open.*

eTable 4. Multilevel Linear Probability Models of the Presence of Any Stigmatizing Language in Admission Notes For Adult Patients Age 18 Years or Older

	**Full Sample**		**Diabetes**		**Substance Use**		**Chronic Pain**	
Predictors[a]	*Estimates*	*CI*	*Estimates*	*CI*	*Estimates*	*CI*	*Estimates*	*CI*
(Intercept)	0.0190	0.0119,0.0262	0.0718	0.0408,0.1027	0.0198	-0.0106,0.0502	0.0219	0.0106,0.0333
Patient Age	0.0002	0.0001,0.0003	-0.0001	-0.0005,0.0003	-0.0001	-0.0005,0.0002	-0.0003	-0.0004,-0.0001
Patient Female	-0.0058	-0.0092,-0.0023	-0.0114	-0.0229,0.0001	-0.0068	-0.0167,0.0031	0.0000	-0.0052,0.0053
Patient Non-Hispanic Asian	-0.0039	-0.0126,0.0049	-0.0080	-0.0413,0.0252	-0.0186	-0.0658,0.0287	-0.0046	-0.0258,0.0165
Patient Non-Hispanic Black	0.0092	0.0039,0.0144	0.0211	0.0047,0.0374	0.0179	0.0040,0.0318	0.0118	0.0040,0.0196
Patient Hispanic	0.0086	0.0005,0.0168	0.0149	-0.0119,0.0417	0.0018	-0.0236,0.0272	0.0083	-0.0057,0.0223
Patient Non-Hispanic Other Race[b]	0.0001	-0.0087,0.0090	-0.0012	-0.0340,0.0315	0.0236	-0.0018,0.0490	-0.0085	-0.0235,0.0066
Preferred Language Other than English	-0.0057	-0.0126,0.0011	-0.0104	-0.0319,0.0111	0.0035	-0.0210,0.0280	-0.0065	-0.0187,0.0057
Advanced Practice Provider (APP)	0.0016	-0.0035,0.0067	-0.0097	-0.0266,0.0072	0.0019	-0.0116,0.0154	0.0014	-0.0058,0.0087
Female Provider	-0.0017	-0.0058,0.0024	-0.0002	-0.0131,0.0127	0.0043	-0.0062,0.0148	0.0016	-0.0042,0.0074
Diabetes Severity Index (aDCSI)			0.0123	0.0023,0.0223				
Type 1 Diabetes Mellitus			0.0056	-0.0169,0.0282				
Substance Use Severity					0.0099	0.0004,0.0195		
Substance Use Disorder in Remission					-0.0100	-0.0220,0.0019		
**Random Effects**								
σ^2	0.03		0.07		0.03		0.01	
τ_{00}	0.00 $_{author}$		0.00 $_{author}$		0.00 $_{author}$		0.00 $_{author}$	
ICC	0.01		0.01				0.01	
N	1724 $_{author}$		1191 $_{author}$		1113 $_{author}$		1042 $_{author}$	
Observations	39224		8032		5627		4715	

[a] Reference Categories: patient male, patient non-Hispanic White, physician provider, type II diabetes mellitus
[b] Non-Hispanic Other Race category includes Native American and Hawaiian/Pacific Islander patients
Advanced practice providers include nurse practitioners, physician assistants, nurse midwives and nurse anesthetists
σ^2 = Within author variance, τ_{00} = Between author variance
ICC=Interclass correlation coefficient

© 2022 Himmelstein G et al. *JAMA Network Open.*

eTable 5. Significant Log Odds Ratios, Odds Ratios, and Weighted Log Odds Ratios of Words in Notes About Non-Hispanic Black vs Non-Hispanic White Patients

Word	Log Odds Ratio	Odds Ratio	Weighted[1] Log Odds Ratio (Z-Score)
**Full Sample**			
nonadherent	2.22	9.17	4.23
abuses	2.06	7.86	2.20
belligerent	1.66	5.24	3.18
cheat	1.37	3.93	2.31
secondary gain	1.25	3.49	1.97
adherence	1.19	3.28	4.25
noncompliance	1.19	3.27	2.38
argumentative	1.15	3.14	2.37
unwilling	0.96	2.62	3.76
compliance	0.91	2.48	3.96
abuser	0.83	2.29	3.10
uncontrolled	0.75	2.11	2.93
refused	0.70	2.01	2.98
drug seeking	0.61	1.83	3.16
abuse	0.44	1.56	3.65
malingering	0.27	1.31	2.14
refuses	0.22	1.25	2.97
difficult patient	0.05	1.05	3.03
**Diabetes**			
unwilling	1.54	4.64	3.30
refused	0.96	2.60	3.17
noncompliance	0.81	2.24	2.82
uncontrolled	0.71	2.04	2.11
refuses	0.15	1.16	3.25
fail	-0.75	0.47	2.23
**Substance Use**			
abuser	1.21	3.36	2.56
narcotics	0.17	1.19	3.09
belligerent	0.70	2.02	2.23
combative	-0.30	0.74	2.04
**Chronic Pain**			
drug seeking	0.32	1.37	2.50
narcotics	0.20	1.22	2.44

[1] Weighted log odds take into account that the (log) odds ratios of infrequently used words may represent random differences rather than true patterns. The weighted log odds ratio should be interpreted as a z-score, with values > 1.96 indicating a significant (*P*<.05) difference in word usage in notes about non-Hispanic Black vs. non-Hispanic White patients

© 2022 Himmelstein G et al. *JAMA Network Open.*

eTable 6. Multilevel Linear Probability Models of the Presence of Any Stigmatizing Language in Admission Notes Written by Physicians in the Full Sample and in Each of 3 Conditions

	**Full Sample**		**Diabetes**		**Substance Use**		**Chronic Pain**	
Predictors[a]	*Estimates*	*CI*	*Estimates*	*CI*	*Estimates*	*CI*	*Estimates*	*CI*
(Intercept)	0.0147	0.0076,0.0217	0.0877	0.0523,0.1231	0.0215	-0.0131,0.0561	0.0182	0.0054,0.0310
Patient Age	0.0003	0.0002,0.0003	-0.0002	-0.0007,0.0003	-0.0004	-0.0007,-0.0000	-0.0002	-0.0003,0.0000
Patient Female	-0.0019	-0.0053,0.0014	-0.0098	-0.0228,0.0032	-0.0098	-0.0207,0.0011	-0.0003	-0.0062,0.0056
Patient Non-Hispanic Asian	-0.0115	-0.0199,-0.0031	-0.0078	-0.0451,0.0296	-0.0408	-0.0903,0.0086	-0.0054	-0.0281,0.0173
Patient Non-Hispanic Black	0.0063	0.0011,0.0114	0.0215	0.0035,0.0395	0.0195	0.0046,0.0344	0.0089	0.0004,0.0173
Patient Hispanic	0.0020	-0.0060,0.0100	0.0156	-0.0145,0.0457	0.0056	-0.0226,0.0338	0.0041	-0.0115,0.0198
Patient Non-Hispanic Other Race[b]	-0.0041	-0.0127,0.0045	0.0077	-0.0279,0.0432	-0.0056	-0.0336,0.0224	-0.0086	-0.0248,0.0077
Preferred Language Other than English	0.0048	-0.0018,0.0115	-0.0084	-0.0326,0.0157	0.0208	-0.0058,0.0473	-0.0098	-0.0234,0.0038
Female Physician	0.0013	-0.0023,0.0050	-0.0025	-0.0162,0.0112	0.0106	-0.0004,0.0217	0.0016	-0.0043,0.0076
Physician PGY	-0.0005	-0.0009,-0.0001	-0.0020	-0.0039,-0.0002	-0.0000	-0.0016,0.0015	-0.0004	-0.0012,0.0005
Diabetes Severity Index (aDCSI)			0.0090	-0.0023,0.0202				
Type 1 Diabetes Mellitus			0.0095	-0.0156,0.0346				
Substance Use Severity					0.0140	0.0033,0.0247		
Substance Use Disorder in Remission					-0.0118	-0.0248,0.0011		
**Random Effects**								
σ^2	0.03		0.07		0.03		0.01	
τ_{00}	0.00 $_{author}$		0.00 $_{author}$		0.00 $_{author}$		0.00 $_{author}$	
ICC	0.00		0.01		0.00		0.00	
N	1822 $_{author}$		1019 $_{author}$		932 $_{author}$		884 $_{author}$	
Observations	39901		6485		4589		3675	

[A] Reference Categories: patient male, patient Non-Hispanic White, physician provider, type II diabetes mellitus
[B] Non-Hispanic Other Race category includes Native American and Hawaiian/Pacific Islander patients
PGY=Post Graduate Year
aDCSI= adapted diabetes complication severity index
σ^2 = Within author variance, τ_{00} = Between author variance
ICC = interclass correlation coefficient, which indicates that share of the variance explained by single providers who authored multiple notes

© 2022 Himmelstein G et al. *JAMA Network Open.*

eTable 7. Multilevel Linear Probability Models of the Presence of Any Stigmatizing Language in Admission Note in the Full Sample and in Each of 3 Conditions, with Interaction Terms

	**Full Sample**		**Diabetes**		**Substance Use**		**Chronic Pain**	
Predictors[a]	*Estimates*	*CI*	*Estimates*	*CI*	*Estimates*	*CI*	*Estimates*	*CI*
(Intercept)	0.0113	0.0049,0.0178	0.0711	0.0399,0.1022	0.0288	-0.0019,0.0594	0.0194	0.0083,0.0306
Patient Age	0.0003	0.0002,0.0004	-0.0001	-0.0005,0.0004	-0.0004	-0.0007,-0.0001	-0.0002	-0.0004,-0.0001
Patient Female	-0.0020	-0.0054,0.0013	-0.0115	-0.0230,0.0001	-0.0112	-0.0211,-0.0013	0.0013	-0.0038,0.0064
Patient Non-Hispanic Asian	-0.0092	-0.0187,0.0003	-0.0068	-0.0472,0.0336	-0.0145	-0.0683,0.0393	-0.0059	-0.0282,0.0164
Patient Non-Hispanic Black	0.0071	0.0018,0.0124	0.0261	0.0091,0.0431	0.0221	0.0080,0.0363	0.0105	0.0027,0.0183
Patient Hispanic	0.0031	-0.0081,0.0143	0.0053	-0.0395,0.0502	0.0084	-0.0231,0.0398	0.0182	-0.0025,0.0390
Patient Non-Hispanic Other Race[b]	-0.0037	-0.0127,0.0054	0.0034	-0.0315,0.0384	0.0151	-0.0116,0.0419	-0.0093	-0.0245,0.0059
Preferred Language Other than English	0.0096	-0.0006,0.0197	0.0042	-0.0260,0.0343	0.0239	-0.0146,0.0624	-0.0075	-0.0240,0.0089
Patient Non-Hispanic Black*Preferred Language Other than English	-0.0083	-0.0292,0.0127	-0.0637	-0.1240,-0.0035	-0.0174	-0.0945,0.0598	-0.0097	-0.0459,0.0266
Patient Hispanic*Preferred Language Other than English	-0.0059	-0.0228,0.0110	-0.0013	-0.0596,0.0569	-0.0118	-0.0701,0.0465	-0.0171	-0.0460,0.0118
Patient Non-Hispanic Asian*Preferred Language Other than English	-0.0133	-0.0341,0.0075	-0.0162	-0.0893,0.0569	-0.0413	-0.1563,0.0737	0.0082	-0.0507,0.0670
Patient Non-Hispanic Other Race*Preferred Language Other than English	-0.0054	-0.0331,0.0222	-0.0451	-0.1449,0.0547	0.0003	-0.0851,0.0856	0.0109	-0.0457,0.0675
Advanced Practice Provider (APP)	0.0018	-0.0030,0.0066	-0.0096	-0.0266,0.0073	0.0018	-0.0121,0.0158	-0.0012	-0.0083,0.0058
Female Provider	0.0002	-0.0037,0.0041	-0.0001	-0.0130,0.0128	0.0074	-0.0033,0.0181	0.0023	-0.0034,0.0080
Diabetes Severity Index (aDCSI)			0.0118	0.0018,0.0218				
Type 1 Diabetes Mellitus			0.0052	-0.0174,0.0278				
Substance Use Severity					0.0114	0.0018,0.0209		
Substance Use Disorder in Remission					-0.0104	-0.0224,0.0015		
**Random Effects**								
σ^2	0.03		0.07		0.03		0.01	
τ_{00}	0.00 $_{author}$		0.00 $_{author}$		0.00 $_{author}$		0.00 $_{author}$	
ICC	0.00		0.01		0.01		0.01	
N	1835 $_{author}$		1191 $_{author}$		1113 $_{author}$		1043 $_{author}$	
Observations	40098		8032		5627		4716	

[a] Reference Categories: patient male, patient Non-Hispanic White, physician provider, type II diabetes mellitus
[b] Non-Hispanic Other Race category includes Native American and Hawaiian/Pacific Islander patients
Advanced practice providers include nurse practitioners, physician assistants, nurse midwives and nurse anesthetists
aDCSI= adapted diabetes complication severity index
σ^2 = Within author variance, τ_{00} = Between author variance
ICC = interclass correlation coefficient, which indicates that share of the variance explained by single providers who authored multiple notes

© 2022 Himmelstein G et al. *JAMA Network Open.*

eTable 8. Falsification Analysis—Multilevel Linear Probability Models of the Presence of Any Alternative to Stigmatizing Language in Admission Note in the Full Sample and in Each of 3 Conditions

	**Full Sample**		**Diabetes**		**Substance Use**		**Chronic Pain**	
Predictors[a]	*Estimates*	*CI*	*Estimates*	*CI*	*Estimates*	*CI*	*Estimates*	*CI*
(Intercept)	0.0480	0.0383,0.0578	0.0945	0.0560,0.1331	0.0266	0.0047,0.0485	0.1204	0.0897,0.1512
Patient Age	0.0002	0.0001,0.0004	0.0002	-0.0003,0.0008	-0.0004	-0.0006,-0.0001	-0.0009	-0.0014,-0.0005
Patient Female	-0.0025	-0.0075,0.0024	0.0013	-0.0131,0.0156	-0.0062	-0.0133,0.0009	-0.0041	-0.0182,0.0101
Patient Non-Hispanic Asian	-0.0076	-0.0199,0.0048	0.0014	-0.0399,0.0426	-0.0113	-0.0449,0.0224	-0.0477	-0.1049,0.0096
Patient Non-Hispanic Black	-0.0022	-0.0098,0.0053	0.0052	-0.0151,0.0255	-0.0041	-0.0140,0.0059	0.0005	-0.0206,0.0217
Patient Hispanic	-0.0040	-0.0158,0.0078	0.0269	-0.0063,0.0602	-0.0082	-0.0264,0.0100	-0.0029	-0.0407,0.0349
Patient Non-Hispanic Other Race[b]	-0.0061	-0.0186,0.0065	0.0070	-0.0337,0.0476	0.0117	-0.0065,0.0298	-0.0060	-0.0465,0.0346
Preferred Language Other than English	-0.0037	-0.0135,0.0060	0.0029	-0.0237,0.0296	0.0111	-0.0063,0.0286	-0.0173	-0.0503,0.0156
Advanced Practice Provider (APP)	-0.0044	-0.0122,0.0034	0.0059	-0.0158,0.0276	-0.0106	-0.0215,0.0003	-0.0208	-0.0413,-0.0004
Female Provider	0.0022	-0.0040,0.0083	-0.0057	-0.0221,0.0107	0.0047	-0.0035,0.0129	0.0155	-0.0008,0.0317
Diabetes Severity Index (aDCSI)			0.0040	-0.0084,0.0165				
Type 1 Diabetes Mellitus			0.0008	-0.0271,0.0288				
Substance Use Severity					0.0078	0.0010,0.0147		
Substance Use Disorder in Remission					-0.0104	-0.0189,-0.0019		
**Random Effects**								
σ^2	0.05		0.10		0.02		0.06	
τ_{00}	0.00 $_{author}$		0.00 $_{author}$		0.00 $_{author}$		0.00 $_{author}$	
ICC	0.01		0.02		0.03		0.02	
N	1835 $_{author}$		1191 $_{author}$		1113 $_{author}$		1043 $_{author}$	
Observations	40098		8032		5627		4716	

[a] Reference Categories: patient male, patient Non-Hispanic White, physician provider, type II diabetes mellitus
[b] Non-Hispanic Other Race category includes Native American and Hawaiian/Pacific Islander patients
Advanced practice providers include nurse practitioners, physician assistants, nurse midwives and nurse anesthetists
aDCSI= adapted diabetes complication severity index
σ^2 = Within author variance, τ_{00} = Between author variance
ICC = interclass correlation coefficient, which indicates that share of the variance explained by single providers who authored multiple notes

© 2022 Himmelstein G et al. *JAMA Network Open.*

eFigure. Log Odds Ratios[a] (Non-Hispanic Black Individuals/Non-Hispanic White Individuals)[b] For WordStems of Stigmatizing Language, in Whole Sample and by Condition

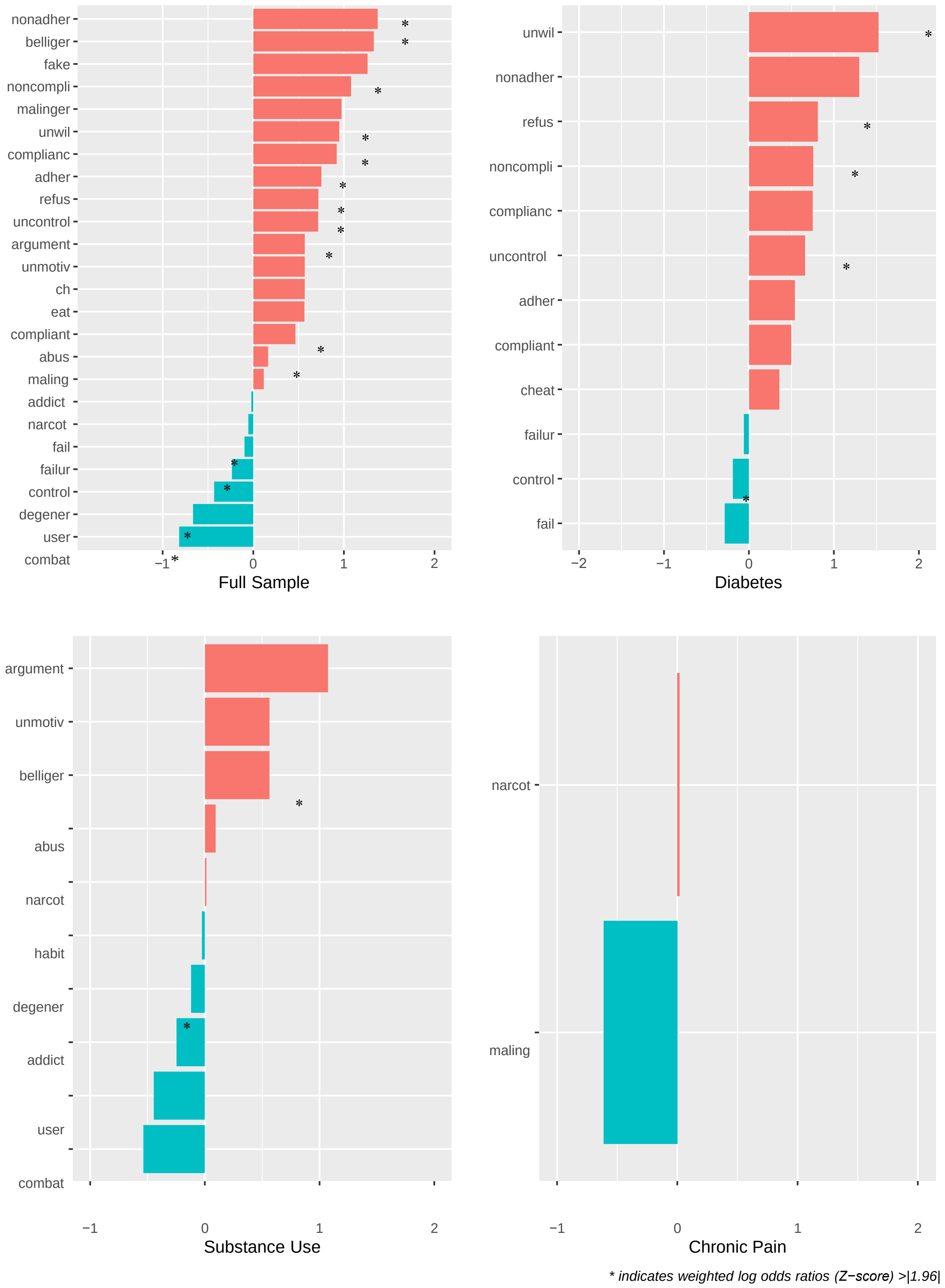

[a] * indicates weighted log odds ratios (Z−score) >|1.96|

[b] Red bars (log odds ratios > 0) indicate language more commonly found in notes about non-Hispanic black patients. Blue bars (log odds ratios < 0) indicate language more commonly found in notes about non-Hispanic white patients.

© 2022 Himmelstein G et al. *JAMA Network Open.*
